# Identification of MiRNA from Eggplant (*Solanum melongena* L.) by Small RNA Deep Sequencing and Their Response to *Verticillium dahliae* Infection

**DOI:** 10.1371/journal.pone.0072840

**Published:** 2013-08-27

**Authors:** Liu Yang, Dengwei Jue, Wang Li, Ruijie Zhang, Min Chen, Qing Yang

**Affiliations:** College of Life Sciences, Nanjing Agricultural University, Nanjing, P.R. China; Virginia Tech, United States of America

## Abstract

MiRNAs are a class of non-coding small RNAs that play important roles in the regulation of gene expression. Although plant miRNAs have been extensively studied in model systems, less is known in other plants with limited genome sequence data, including eggplant (*Solanum melongena* L.). To identify miRNAs in eggplant and their response to *Verticillium dahliae* infection, a fungal pathogen for which clear understanding of infection mechanisms and effective cure methods are currently lacking, we deep-sequenced two small RNA (sRNA) libraries prepared from mock-infected and infected seedlings of eggplants. Specifically, 30,830,792 reads produced 7,716,328 unique miRNAs representing 99 known miRNA families that have been identified in other plant species. Two novel putative miRNAs were predicted with eggplant ESTs. The potential targets of the identified known and novel miRNAs were also predicted based on sequence homology search. It was observed that the length distribution of obtained sRNAs and the expression of 6 miRNA families were obviously different between the two libraries. These results provide a framework for further analysis of miRNAs and their role in regulating plant response to fungal infection and Verticillium wilt in particular.

## Introduction

Gene expression in plants is highly regulated to ensure proper development and appropriate responses to environmental changes. As gene expression is a multi-step process, it can be regulated at several levels. One of the most important regulatory mechanisms is post-transcriptional regulation, which involves 21–24 nucleotide (nt) small RNA (sRNA) molecules [1]. The sRNA content of plant cells is surprisingly complex, suggesting an extensive regulatory role for these molecules [2]. Distinguished by their origin and biological function, several classes of small regulatory RNAs have been identified. One of these is small interfering RNAs (siRNAs), which encompasses chromatin-associated siRNAs, trans-acting siRNAs (tasiRNAs), repeat-associated siRNAs (rasiRNAs), and natural antisense transcript-associated siRNAs (nat-siRNAs) [3]. All these siRNAs derive from double-stranded RNA (dsRNA), but dsRNA can be formed through different mechanisms. MiRNAs are generated by DICER-LIKE 1 (DCL1) and/or DCL4 from single stranded (ss) hairpin RNA precursors which can be encoded in their own genes or exist in introns of protein-coding genes [4]. Other endogenous sRNAs are processed from long dsRNA and often require RNA-dependent RNA polymerase 6 (RDR6) (*trans*acting siRNAs) [5], [6], RDR2 (heterochromatin siRNAs) [7], or overlapping antisense mRNAs (natural antisense siRNAs) [8]. It is possible that there are other unidentified mechanisms leading to dsRNA that could be sources of new classes of sRNAs.

The best-characterized class of plant sRNAs is miRNA [9]. MiRNAs are an abundant class of small endogenous RNAs, 20–25 nt in length, that regulate gene expression post-transcriptionally by targeting transcripts for cleavage or translational repression [9]. MiRNA-guided gene silencing is now known as a conserved and essential regulatory mechanism for plant development, metabolism, as well as for adaptation to stress conditions [10]–[12].

Increasing evidence indicates that miRNAs play critical roles in regulating abiotic and biotic stress responses, including disease resistance [13]–[16]. MiR393 was the first miRNA that was found to contribute to plant immune systems. In *Arabidopsis*, miR393 can be induced by bacterial elicitor flg22 and positively contributes to pathogen associated molecular pattern (PAMP)-triggered immunity (PTI) by negatively regulating messenger RNAs for the F-box auxin receptors and subsequently suppressing auxin signaling [15]. It has been recently demonstrated that miR393* also contributes to immunity in *Arabidopsis*, mainly effector-triggered immunity (ETI), by modulating secretion of PR1 [13]. In addition to miR393, miR167 and miR160, which target auxin response factors (ARF) were also induced by a non-pathogenic *Pseudomonas syringae* pv. Tomato (pst) DC3000 strain with a mutated type III secretion system hrcC [17]. In turn, repression of auxin signaling was shown to restrict *P. syringae* growth, implicating auxin in disease susceptibility and miRNA-mediated suppression of auxin signaling in disease resistance [15]. Another recent study reported an endogenous *Arabidopsis* siRNA that is specifically induced by the bacterial pathogen *P. syringae* carrying AvrRpt2 [16]. This siRNA contributes to RPS2-mediated disease resistance by repressing a putative negative regulator of the RPS2 resistance pathway. Positional cloning in an *Arabidopsis* mutant that was susceptible to several pathogens revealed a mutation in the Argonaute gene *ago4*, which is associated with small interfering RNAs involved in RNA-directed DNA methylation (RdDM) [18].

Verticillium wilt is a notorious wilt disease affecting over 300 species of eudicot plants and mainly caused by a soil-borne fungal pathogen, *Verticillium dahliae* Kleb. [19]. Many economically important plants are susceptible to this disease, including eggplants (*Solanum melongena* L.). As a common vegetable crop, eggplant suffers constantly from *Verticillium* infection, causing significant economic losses, but there remain at present no proven cures (chemical or cultural) for this disease since the mechanisms of Verticillium wilt remain poorly understood. Some recent studies on *Arabidopsis* mutants have tested the possible role of RNA-mediated gene silencing in plant defense against *Verticillium* and revealed several components that were shown to affect *Verticillium*-specific defense, suggesting that multiple RNA silencing pathways play significant roles in the regulation of pathogen defense responses [20].

To date, no systematic studies of small RNAs in eggplant have been conducted. In this study, we deep-sequenced two sRNA libraries prepared from mock-infected and *Verticillium dahliae* infected seedlings of eggplants to investigate the miRNAs in eggplant and their transcriptional profile in response to *Verticillium dahliae* infection. Our work will lay the foundation for further analysis and understanding of miRNA function in the regulation of *Verticillium dahliae*-caused defense responses in eggplants.

## Materials and Methods

### Plants, Pathogen and Infection

The eggplant (*Solanum melongena* L.) cultivar Suqi was used in the experiment. Eggplant seedlings were cultured in sugar-free nutrient medium (quarter-strength MS solution) at 25°C under a 16 h light:8 h dark regime. For the NPA (N-1-naphthylphthalamic acid, Sigma-Aldrich), SA (salicylic acid, Sigma-Aldrich) and IAA (3-Indoleacetic acid, Sigma-Aldrich) treatments, seedlings were transferred to tubes containing different concentrations of the chemicals and, 24 h later, they were inoculated with pathogen.

*Verticillium dahliae* isolated from eggplant with Verticillium wilt was kindly provided by the College of Plant Protection, Nanjing Agricultural University (Nanjing, China). The pathogen was cultured on both potato dextrose agar (PDA) plate for 15 days at 25°C to collect spores (at a concentration of 5×10^7^spores·ml^−1^), and in Czapeck’s liquid medium to obtain crude toxin (8 mg·ml^−1^).

Uniform seedlings with five main leaves were infected with a mixture of spores and crude toxin for 12 h (hereafter called TR). Control (mock-inoculated) seedlings were inoculated with water (hereafter called CK). Six uniform seedlings were used in each treatment. Three of them were randomly selected for total RNA extraction and small RNA library preparation. Whole seedlings were employed for RNA extraction. The other three seedlings in each treatment were used to confirm whether the infection was successful. Both the pathogen-infected and mock-infected seedlings were frozen in liquid nitrogen immediately after inoculation.

The number of diseased plants and infected leaves was recorded on the seventh day post-inoculation. Disease intensity was graded using the method of Liu et al. [21] as follows: grade 0, no disease dots on the leaves of the plants; grade 1, one to two withered leaves; grade 2, three to four withered leaves; grade 3, most leaves withered; and grade 4, the plant is withered nearly to death. A disease index was calculated according to the disease grading criterion of grades 0 to 4 using the formula: disease index = [Σ (number of diseased plants × disease grade)/(total number of investigated plants × the highest disease grade)] ×100.

### Small RNA Library Preparation and Sequencing

For direct comparison, eggplant seedlings used for CK and TR library construction were grown under the same conditions except for the pathogen infection. Total RNA was extracted from the above described samples using TRIzol reagent (Invitrogen), and subsequently subjected to 15% denaturing polyacrylamide gel electrophoresis, after which the sRNA fragments of 18–28 nt were isolated from the gel and purified. Next, the sRNA molecules were ligated to a 5′ adaptor and a 3′ adaptor sequentially and then converted to DNA by RT-PCR. Finally, 20 µg of RT-PCR product was sequenced directly using an Illumina/Solexa 1 G Genome Analyzer according to the manufacturer’s protocols (BGI, Shenzhen, China). The obtained sequence libraries were subjected to Illumina/Solexa’s sequencing-by-synthesis method. The two constructed cDNA libraries underwent Illumina/Solexa’s proprietary flowcell cluster generation and bridge amplification. After which the 1 G sequencer, during 36 cycles of extension, recorded fluorophore excitation and determined the sequence of bases for each cluster. After image analysis, sequence quality evaluation and summarization of data production were performed with Illumina/Solexa Pipeline.

### Small RNA Analysis

After removing the adaptor/acceptor sequences, filtering low quality tags and cleaning up contamination due to adaptor-adaptor ligation, the occurrences of each unique sequence read were counted as sequence tags. BLAST searching against eggplant ESTs (about 98,089 ESTs) was performed using SOAP 2.0 [22]. All these sequence tags were compared with the sequences of non-coding RNAs (rRNA, tRNA, snRNA, snoRNA) available in Rfam (http://www.sanger.ac.uk/software/Rfam) [23] and the GenBank noncoding RNA database (http://www.ncbi.nlm.nih.gov) to classify degradation fragments of noncoding RNA. The remainder of the sequences which matched eggplant ESTs were searched for miRNA sequences using miRBase 19 (http://www.mirbase.org/index.shtml) [24] to identify the known miRNAs by allowing either shorter/longer or containing up to two mismatches. Subsequently, we performed extensive comparisons against known miRNAs in other plant species to investigate the evolutionary conservation of known miRNAs in eggplant versus other plants.

### Prediction of Novel miRNA

After searching against the Rfam, NCBI GenBank, and miRBase databases, the remaining sequences that were not associated with any annotated type were used to map the ESTs for prediction of novel miRNA candidates. Prediction of eggplant miRNAs was conducted using previously developed criteria [25]. MiRNA precursors have characteristic fold-back structures that can be used to predict novel miRNAs. The prediction was implemented in the Mireap program developed by the BGI (Shenzhen, China). To identify atypical and novel sequences of miRNAs in eggplant, we adopted the following strategy. First, candidate miRNA sites were screened out from breakpoints defined by mapping of the sRNAs. Next, a minimal stringent criterion was used to select miRNA candidates, which ensured that the majority of sequences recovered were known miRNAs. Finally, RNA secondary structure was checked using Mfold [26].

### Prediction of miRNA Targets

The identified known miRNAs and predicted novel miRNAs were used to query sequences for target sites on the psRNAtarget web server (http://biocomp5.noble.org/psRNATarget/). Target transcripts containing complementary sequences of miRNAs were determined with previously established criteria [27]–[29]. Functional categories of obtained EST sequences were annotated against the COG database (http://www.ncbi.nih.gov/COG/) using BLAST with a cutoff of E value <1e-5.

### Differential Expression of Known miRNA

For investigation of Verticillium wilt-responsive miRNAs, the read counts of the identified known miRNAs in both of the two libraries were first normalized to the total number of miRNA reads in each given sample and multiplied by a million. Bayesian methods were then applied to infer statistical significance [30]. MiRNAs with normalized read values less than 1 in both libraries were filtered from further expression analysis because low expression level tends to cause false results. If the normalized read counts of miRNAs in the TR library differed from those in the control CK library, and the p-value of the chi-square test was less than 0.05, this miRNA was considered a Verticillium wilt-responsive miRNA [12] The absolute value of |log_2_^Ratio^|>1 was used as the threshold to judge the statistical significance of miRNA expression.

### Confirmation of Mature miRNAs and their Targets Expression

Expression profiles of mature miRNAs and their target genes were assayed by RT-PCR and performed as described previously [31]. The primers used for stem-loop reverse transcription and other PCR programs were designed following previously described methods [31] and are listed in Table S1. To standardize the results, the relative abundance of U6 and *EF-1α* were used as the internal standard for miRNAs and target genes respectively.

## Results

### High-throughput Sequencing of Eggplant Small RNAs

Two sRNA libraries prepared from mock-infected (CK) and *Verticillium dahliae*-infected (TR) eggplants were sequenced by Illumina/Solexa, a high throughput sequencing technology producing highly accurate, reproducible and quantitative readouts of sRNAs [32], [33], which can be used as a tool for miRNA expression profiling [34]–[36]. The sequencing data were deposited at Gene Expression Omnibus (accession number: GSE46330).

Illumina/Solexa sequencing of CK and TR libraries generated a total of 10,364,226 and 20,758,478 raw reads, respectively. After filtering, 10,258,739 clean reads in the CK library and 20,572,053 clean reads in the TR library ranging from 18 to 30 nt were obtained, representing 2,759,845 and 5,973,124 unique sequences, respectively (Table 1). Since the eggplant genome is unknown, BLAST searching against eggplant ESTs (about 98,089 ESTs) was performed, resulting in a total of 256,426 (2.50%) sequences in the CK library and 706,892 (3.44%) sequences in the TR library that could match at least one EST. 33,400 unique sequences from the CK library and 59,481 unique sequences from the TR library were similar to known miRNAs from other plant species that had previously been deposited in miRBase 19. Annotation of rRNAs, scRNAs, snoRNAs, snRNAs and tRNAs was carried out by BLASTn to NCBI Genbank and Rfam databases (Table 1).

**Table 1:** Statistics of small RNA sequences from CK and TR libraries of eggplant.

	Total reads	Percent (%)	Unique reads	Percent (%)
CK				
Raw reads	10,364,226			
Clean reads (18–30 nt sRNA)	10,258,739	100%	2,759,845	100%
miRNA	3,133,527	30.54%	33,400	1.21%
rRNA/tRNA/snRNA/snoRNA	648,545	6.33%	42,650	1.55%
Un-annotation	6,476,661	63.13%	2,683,789	97.24%
sRNA mapping to genome	256,426	2.50%	21,906	0.79%
TR				
Raw reads	20,758,478			
Clean reads (18–30 nt sRNA)	20,572,053	100%	5,973,124	100%
miRNA	4,395,136	21.36%	59,481	1.00%
rRNA/tRNA/snRNA/snoRNA	1,604,454	7.8%	88,656	1.49%
Un-annotation	14,572,433	70.84%	5,824,970	97.52%
sRNA mapping to genome	706,892	3.44%	36,619	0.61%

**Figure 1:**
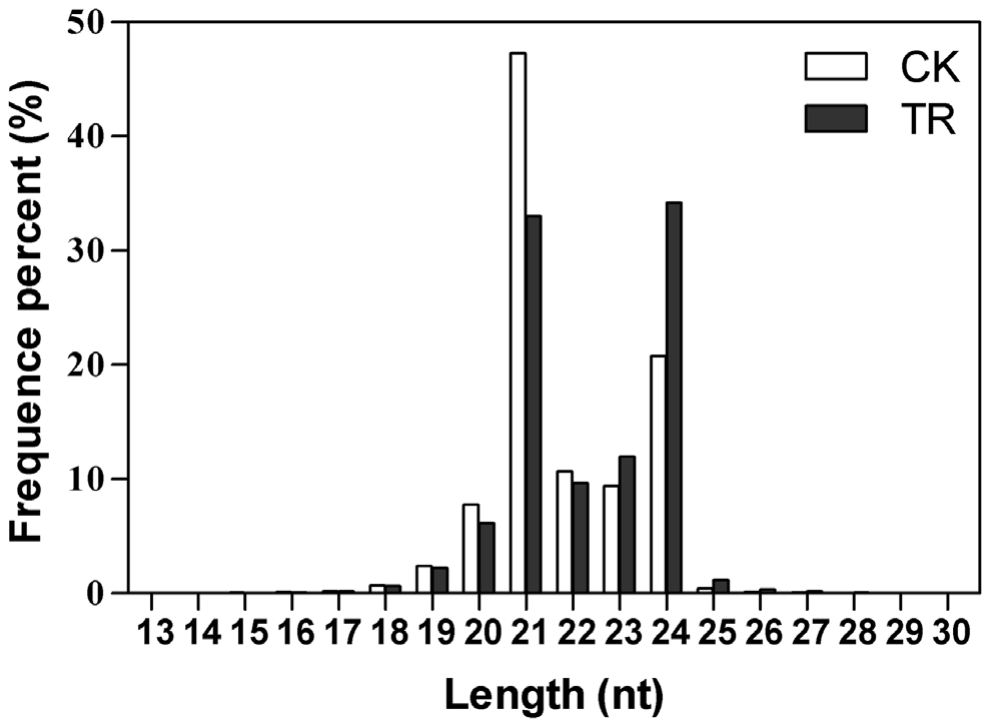
Size distribution of eggplant small RNAs in CK and TR libraries.

**Figure 2:**
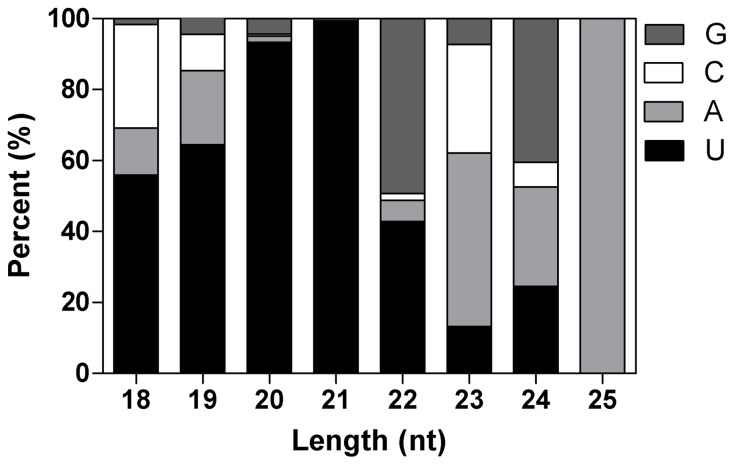
First nucleotide bias of CK library.

The majority of the obtained sRNA sequences from the two libraries were 20–24 nt in size, which is the typical size range for Dicer-derived products (Figure 1). In the CK library, most miRNA sequences, especially those 21-nt long, start with uridine (U), similar to previous results from other plants. However, the majority of 23-nt and 24-nt siRNAs have adenosines (A) and guanine (G) as their 5′ first nucleotide respectively (Figure 2), which differs from some previous studies [37]–[39]. The same trends were also observed in the TR library.

### Known miRNAs and Evolutionary Conservation

Since miRNAs have been shown to play critical roles in many aspects of plant responses to biotic and abiotic stress, we compared our combined (CK and TR libraries) sRNA dataset to known miRNAs in miRBase 19 to analyze the presence of miRNAs in eggplant. Although 5,940 plant miRNAs have been identified in miRBase 19, only 220 miRNAs belong to Solanaceae, which include 165 miRNAs of *Nicotiana tabacum*, 44 miRNAs of *Solanum lycopersicum*, 11 miRNAs of *Solanum tuberosum*, and no available data for eggplant. Among the 30,830,792 clean reads obtained by deep sequencing in both libraries, we found 3,306 sequences from the CK library and 4,255 sequences from the TR library matching 99 known miRNA families (Table S2).

In order to investigate the evolutionary roles of the identified known miRNAs, we performed extensive comparisons against known miRNAs in other plant species, including *Nicotiana tabacum*, *Solanum lycopersicum*, *Solanum tuberosum*, *Arabidopsis thaliana*, *Chlamydomonas reinhardtii*, *Pinus taeda*, *Physcomitrella patens*, *Medicago truncatula*, *Glycine max*, *Gossypium hirsutum*, *Populus trichocarpa*, *Brassica napus*, *Vitis vinifer*, *Citrus sinensis*, *Malus domestica*, *Cucumis melo*, *Zea mays*, *Oryza sativa*, *Sorghum bicolor* and *Triticum aestivum* (Table S2).

Among the identified known miRNAs of eggplant, 8 out of 99 families were not deeply conserved, as no orthologs were detected in the 20 other plant species used for comparison. 38 conserved miRNA families were shared between eggplant and the other three identified species in Solanaceae (*Nicotiana tabacum*, *Solanum lycopersicum* and *Solanum tuberosum*). 13 of these 38 miRNAs had no orthologs in the other selected plants, indicating that these 13 miRNAs were probably involved in regulation of *Solanaceae*-specific processes. In addition, some miRNAs including miR156, miR160, miR166, miR167 and miR171, which are deeply conserved even in lower plants such as *Physcomitrella patens* [40], and others, including miR158 and miR170, which were considered specific to *Arabidopsis thaliana*, were also found in eggplant.

The sequence counts of miRNAs in the libraries were used for estimating the relative abundance of miRNAs. We analyzed the number of reads for detected miRNAs and found a large divergence in expression. The counts of the identified known miRNAs varied from 1 to 3,062,423, with miR157 family being the most abundant miRNA in both of our two sequencing datasets, accounting for about 17% of the clean reads. As it is a deeply conserved miRNA family detected, miR157 is also abundant in other plant families including Brassicaceae, Solanaceae, Malvaceae and Fabaceae.

### Novel miRNAs Detected in Eggplant

An important feature that distinguishes miRNAs from other sRNAs is the ability of the miRNA flanking sequences to fold back in a hairpin structure [41]. As the eggplant genome remains unknown, we have to rely on EST sequences to predict the hairpin structure. Since sequence information for eggplant is limited, our search for new miRNAs revealed only 2 sequences that perfectly matched eggplant ESTs and were able to fold into stem-loop structures, and were considered as such to be novel miRNAs (Table 2, Figure S1), which we denoted m0001 and m0002. Both of them had miRNA star (miRNA*) sequences that were detected in their libraries, and were both 21 nt in length. According to Mfold (http://mfold.rna.albany.edu/?q=mfold), the negative folding free energies of their precursors were −28.10 and −48.90 kcal/mol, respectively.

**Table 2:** List of putative novel miRNAs in eggplant.

Name	Sequence	Length	Arm	Gene ID	Precursor Length (nt)	Energy (kcal/mol)
m0001	TTTGGTTTGAAAGATTGGGTT	21	5p	DQ206075	107	−28.10
m0002	CTTGTGAAGGTAGAGATAGCA	21	5p	FJ597628	194	−48.90

Searching against eggplant ESTs, m0001 matched a pseudogene, while m0002 matched a protein-coding gene. This pseudogene derived from *I2*, a disease resistance gene, which has been *detected* previously in *many* Solanum *species.* This observation indicated that the putative novel miRNA m0001 identified in this study may be specific to eggplant or to Solanaceae more generally. Whereas the putative novel miRNA m0001 was detected in both CK and TR libraries, m0002 was detected only in the TR library.

### Prediction of miRNA Targets in Eggplant

An important step in understanding the biological functions of miRNAs is the identification of their targets. It was demonstrated previously that plant miRNAs have perfect or near-perfect complementarity to their target-site, allowing effective prediction of the target sequences by computation [42]. A total of 320 putative targets were predicted for 99 identified known miRNAs and two new miRNAs by the web tool psRNATarget (http://plantgrn.noble.org/psRNATarget/) [43] using the *Solanum melongena* (eggplant) DFCI gene index (SOMEGI) release 1 for target search, with a setting of 3 as the maximum expectation (Table S3). Most of these target-sites are located in coding regions as previously reported in plants.

**Figure 3:**
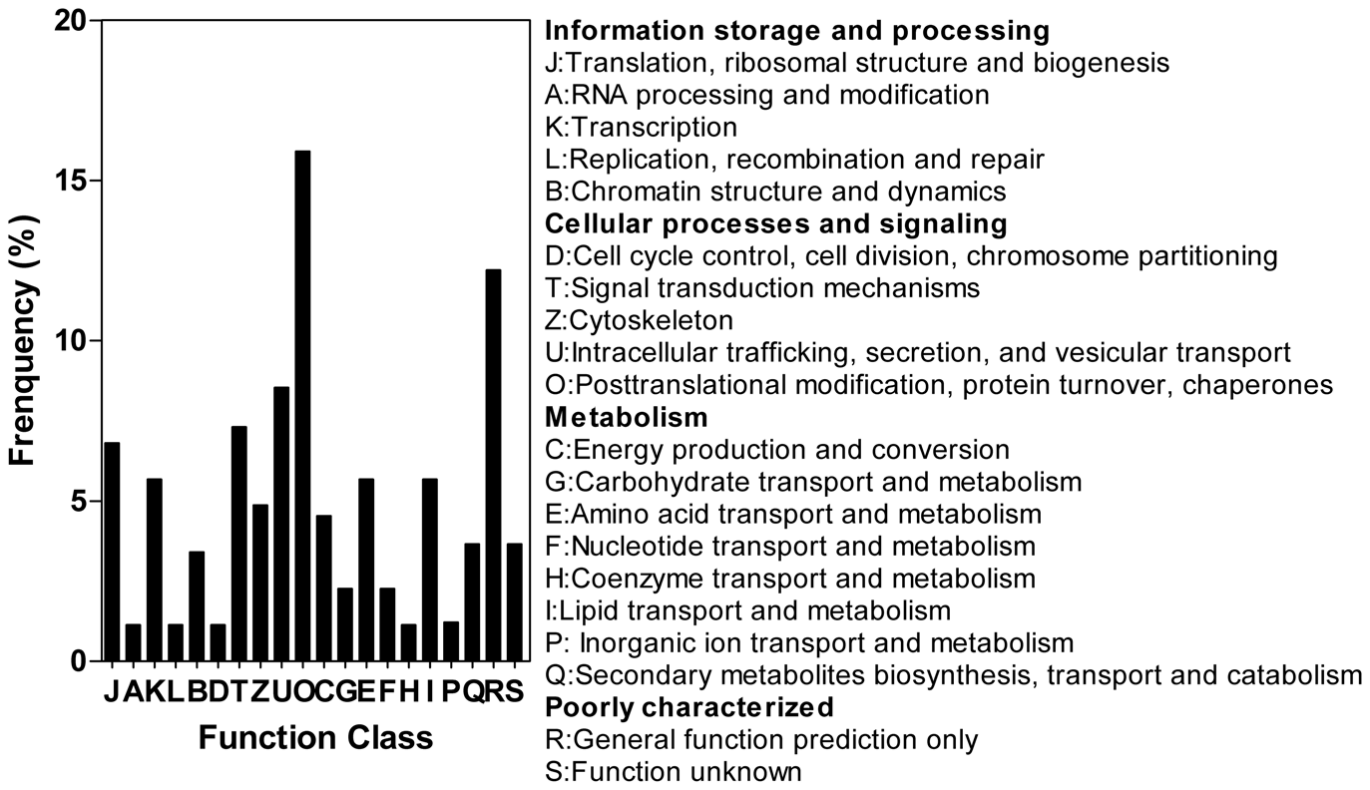
COG function classification of predicted target genes.

All the putative targets were classified into dozens of biological processes according to the COG database (Figure 3). As shown in Figure 3, more than one- third of the target genes were involved in cellular processes and signaling, while more than 15% of the target genes were involved in post-translational modification. We also determined that 15.9% of the predicted targets were poorly characterized genes, suggesting possible new roles for these miRNAs in eggplant. Notably, only three of these predicted targets are transcription factor genes. MiR2950 and miR5284 were predicted to target bZIP transcription factor, which has been reported to play a crucial role in pathogen defense, signaling, seed maturation, and flower development [44].

### Differentially Expressed miRNAs between CK and TR Libraries

To detect which miRNAs were involved in the response to *Verticillium dahliae* infection, we employed Illumina/Solexa technology to examine expression of miRNAs in eggplant seedlings with (TR library) and without (CK library) *Verticillium dahliae* treatment. The two libraries shared 22,666,440 (73.52%) sequences among the total sRNAs representing 1,016,641 (13.18%) unique sRNAs, which indicated that the sequences present in both libraries were more highly expressed than library-specific sequences (Table S4). In these unique sRNAs, the count of TR-specific sRNA was 4,956,483 (64.23%), which is approximately 3-fold higher than CK-specific sRNAs (1,743,204 reads, 22.59%). These library-specific sRNAs showed which miRNAs were expressed in response to *Verticillium dahliae* infection.

We also compared the size distribution of sRNAs between the two libraries. Approximately 75% of total sRNAs were 20–24 nt in length, with modes of 21 and 24 nt, (Figure 1), consistent with being products of cleavage by DCL enzymes. For CK libraries, the sRNA distribution showed a primary mode at 21 nt (47.26%), and a secondary mode at 24 nt (20.75%). Contrastingly, the primary mode in TR was at 24 nt (34.19%), and secondary mode was 21 nt (33.02%). Assuming that the overall amount of 24-nt sRNA is related to the extent of transcriptional regulation, and given that longer sRNAs are often associated with DNA methylation and heterochromatin formation, this observation suggests more extensive regulation of gene expression by sRNAs at the transcriptional level in TR versus CK.

**Figure 4:**
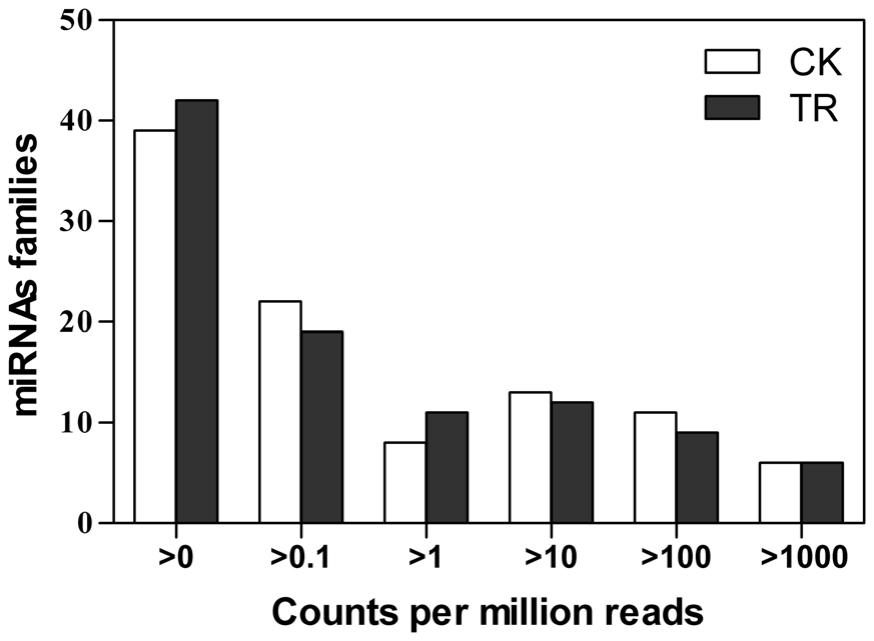
Distribution of miRNA counts over different tag abundance categories from CK and TR library.

Expression of miRNAs spanned a very broad range which varied from several reads to several hundred thousand reads between libraries. MiRNA expression abundance in data sets was analyzed by counting the number of transcripts per million (TPM) clean reads in libraries. The distribution of miRNA counts showed similar tendencies for the two libraries (Figure 4). In the total data set, miR156, miR157, miR166, and miR167 had the largest numbers of reads across both libraries.

**Figure 5:**
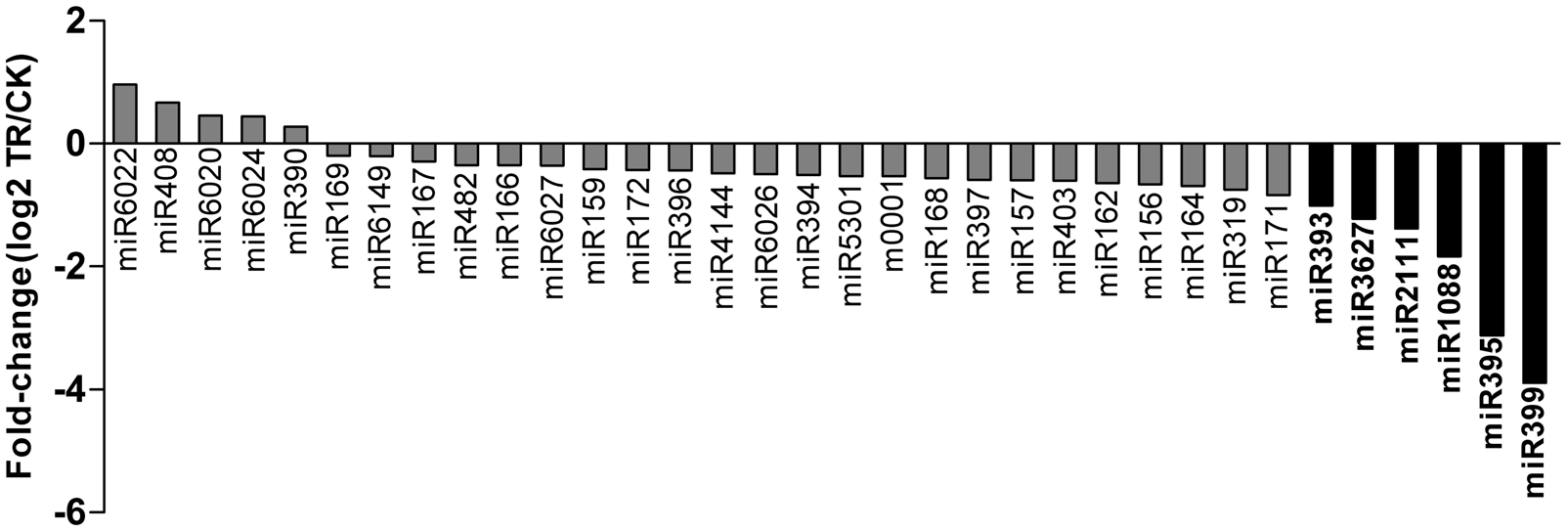
Differential expression of Verticillium wilt-responsive miRNAs. MiRNAs with significantly changed (P<0.05 and |log_2_^Ratio^|>1) expression are highlighted in boldface.

The variation in sequence counts between miRNA families suggests distinct physiological roles in Verticillium wilt. Differentially-expressed miRNAs between libraries are indicative of molecular events involved in responses to *Verticillium dahliae* infection. Overall, 33 miRNAs were identified to be *Verticillium dahliae-*responsive miRNAs (Figure S2 and Table S5). Among these, 28 miRNAs were down-regulated and 5 miRNAs were up-regulated after 12 h treatment (Figure 5), which indicated that the expression levels of many miRNAs were reduced during *Verticillium dahliae* infection. 6 known miRNAs showed significant differential expression (*P*<0.05 and |log_2_^Ratio^|>1) in response to *Verticillium dahliae* infection (Figure 5). Expression levels for all of the significantly changed miRNAs were down-regulated. The most obvious alteration was observed in miR399, whose expression level was about 4-fold lower in the TR library compared to the CK library. Separately, although it has been shown previously that miR393 can be induced by pathogens in *Arabidopsis*, in our study, miR393 decreased significantly in the *Verticillium dahliae*-infected eggplant seedlings. When the eggplant seedlings were exposed to *Verticillium dahliae* for 12 h, many deeply-conserved miRNAs (eg. miR156, miR159, miR160, miR162, miR166, miR167, miR169, miR171, miR172, miR319 and miR396) were all slightly down-regulated, but showed no significant changes. Although the expression of putative novel miRNA m0001 varied from 2,670 in the CK library to 3,708 in the TR library, it did not show a significant change after 12 h of infection by *Verticillium dahliae*. By contrast, the putative miRNA m0002 was detected only in the TR library.

Validation and expression patterns of miRNAs and their target genes in eggplants.

**Figure 6:**
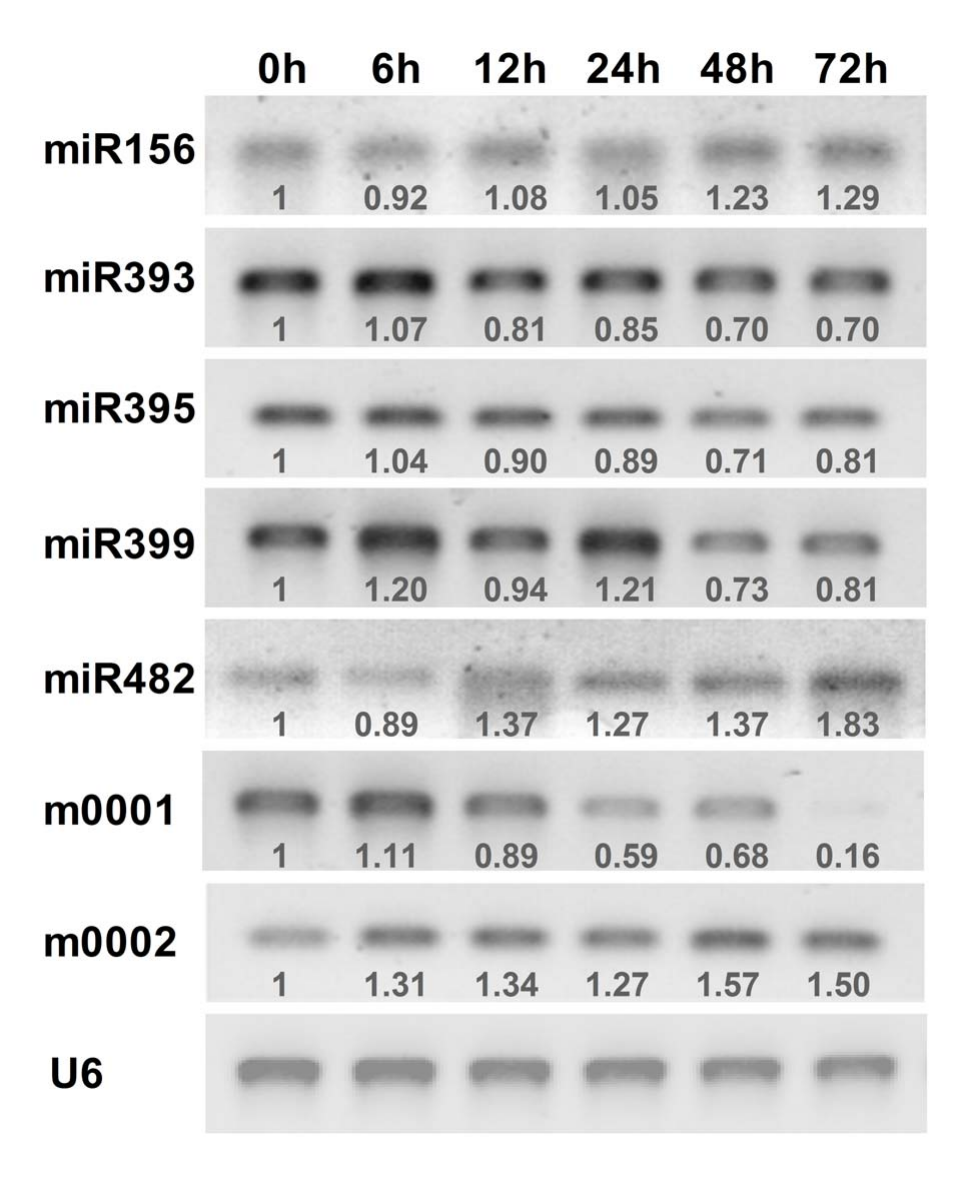
Expression profiles of selected miRNAs in response to *Verticillium dahliae* infection. The number below the band indicates the relative expression level of each gene. Raw values were normalized against loading control U6. The expression level of plant at “0 h” was considered as background level and set to 1.

To confirm the results obtained from small-RNA deep sequencing, we examined the expression patterns of the selected miRNAs and their target transcripts. By using the stem-loop primers in the reverse transcriptase reaction, we measured mature miRNA expression in mock-infected and infected seedlings of eggplants at different times. The stem-loop RT-PCR results of the seven selected miRNAs were similar in magnitude to those obtained by deep sequencing, and confirmed the changes in miRNA expression in response to *Verticillium dahliae* infection (Figure 6). For example, the expression of miR393 continuously decreased after infection with *Verticillium dahliae*, which was consistent with results from the deep sequencing data.

**Figure 7:**
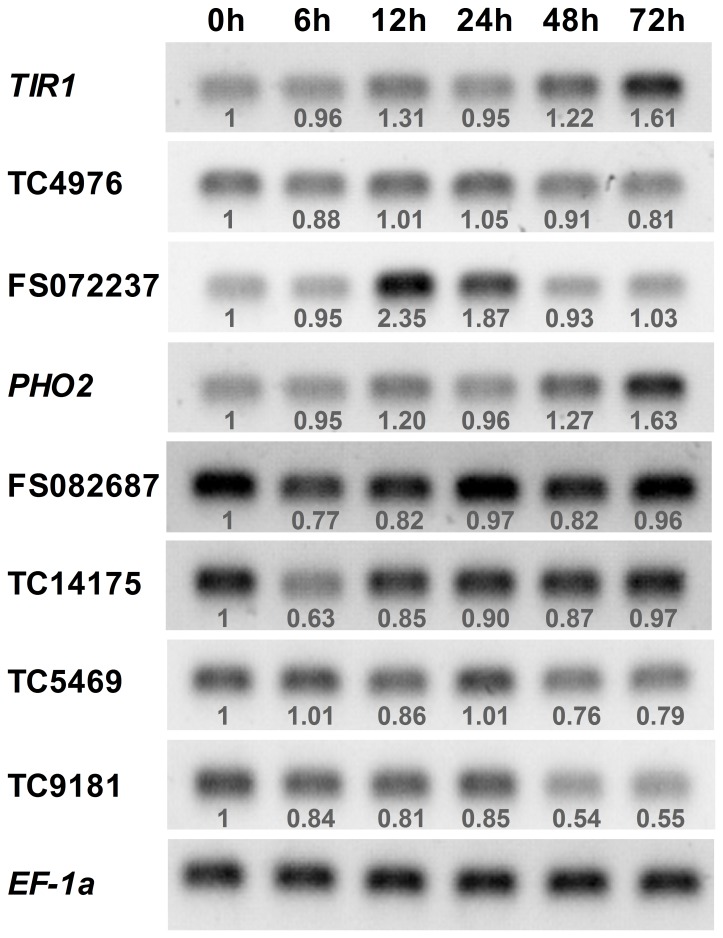
Expression profiles of selected target genes. *TIR1*, the target of miR393; TC4976 and FS072237, targets of miR395; *PHO2*, target of miR399; FS082687 and TC14175, targets of m0001; TC5469 and TC9181, targets of m0002. The number below the band indicates the relative expression level of each gene. The raw values were normalized against loading control *EF-1α*. The expression level of plant at “0 h” was considered as background level and set to 1.

We also examined the expression patterns of eight chosen targets to evaluate if the observed differential expression in miRNA had a direct effect on their target transcript abundance. As shown in Figure 7, miRNA-mediated regulation of target gene expression level appears to be occurring, except for TC4976. This might indicate that TC4976 was not the target of miRNA395, or its expression was also regulated by other factors [35]. Previously, *TIR1* and *PHO2* genes had been identified as targets of miR393 and miR399, respectively. Figure 7 shows that, in most cases, *TIR1* and *PHO2* levels are negatively regulated by their corresponding miRNAs during *Verticillium dahliae* infection.

### Pharmacological Inhibition of Auxin Transport Leads to Increased *Verticillium dahliae* Infection

**Figure 8:**
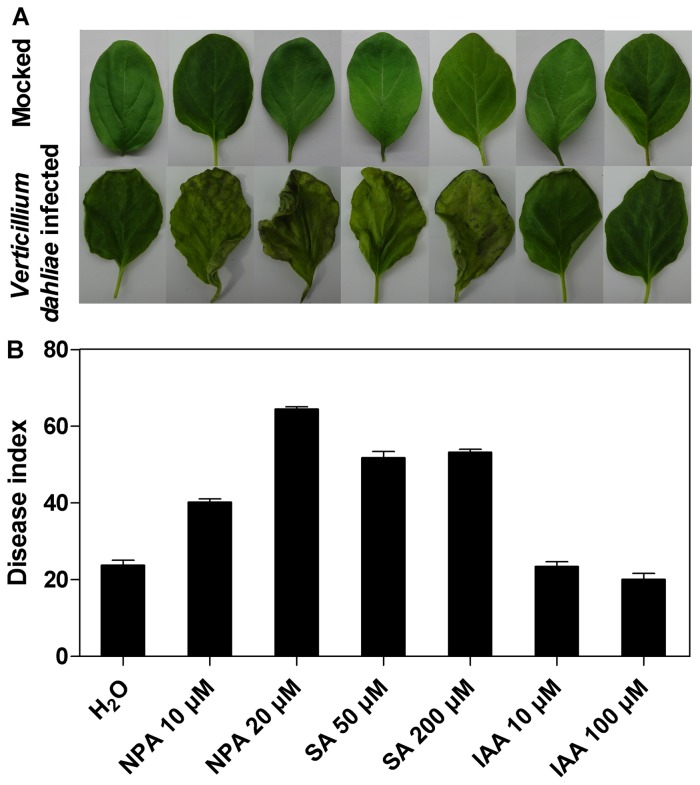
Effects of NPA, SA and IAA on eggplant response to *Verticillium dahliae* infection. Eggplant seedlings with five main leaves were pretreated with different concentrations of NPA, SA, or IAA for 24 h and then infected with *Verticillium dahliae* for 7 days. Representative leaves were subsequently photographed (A) and the disease index was calculated (B).

The changes in miR393 expression observed here point to a general transcriptional induction of the auxin response to *Verticillium dahliae* infection. To test this hypothesis, we pretreated eggplant seedlings with different concentrations (10 and 20 µM) of the auxin transport inhibitor N-1-naphthylphthalamic acid (NPA) [45] for 24 h. Plants were then mock-inoculated and inoculated with *Verticillium dahliae*. Susceptibility was determined after 7 d of treatment. As expected, the disease index was higher in seedlings pretreated with 10 and 20 µM NPA than seedlings without NPA pretreatment (Figure 8). Moreover, there was no difference between seedlings pretreated with NPA alone and control group. In previous studies, salicylic acid (SA) was reported to inhibit pathogen growth in plants through repression of auxin signaling pathway [46]. Thus we also used different concentrations of SA (50 and 200 µM) to inhibit auxin signaling pathway. Similar results were also observed. Seedlings pretreated with SA exhibited enhanced susceptibility to *Verticillium dahliae* infection compared to un-pretreated seedlings. These results indicate that reduced miRNA393 expression in response to *Verticillium dahliae* infection might be a protective response in plants. We also tested the effect of exogenous treatment with different concentrations (10 and 100 µM) of auxin before *Verticillium dahliae* inoculation, but only slight changes in plant susceptibility were observed.

## Discussion

### Evolutionary Conservation of miRNA in Eggplant

MiRNAs are a group of small non-coding RNAs that play important roles in various developmental and stress response processes through negatively regulation of gene expression [47]. MiRNAs have been identified experimentally in many plant species, especially in model plants. However, to our knowledge, only six miRNAs have been identified in *Solanum melongena* (brinjal, eggplant) using bioinformatic methods [48], and no experimental studies have been performed on identifying and analyzing miRNAs in eggplant. In this study, the investigation of entire sets of sRNAs was performed with high-throughput sequencing technology and their response to *Verticillium dahliae* infection was also analyzed, providing useful information to deepen our understanding of the function and regulatory mechanisms of miRNAs in *Verticillium*-defense response. Our study revealed the existence of 99 known miRNA families as well as 2 new predicted miRNAs by Illumina/Solexa technology, which is very useful in investigating miRNA expression profiles [49]. Also, we identified a number of miRNAs which were previously described as species/lineage-specific miRNAs.

To date, more than 21 miRNA families have been found in more than 20 plant species, and they are conserved between dicots and monocots, as well as in mosses [50]. Well-conserved miRNAs often retain homologous target interactions and perform analogous molecular functions across phyla over evolutionary time [51]. For instance, many previous studies have shown that the most conserved miRNAs (eg. miR156, miR159, miR164, miR166, miR167, miR169, miR171, miR172, miR319 and miR396) directly regulate their target mRNAs which encode diverse families of transcription factors such as TCPs, ARFs, MYBs, SPLs, NACs, HD-ZIPs, SCLs, GRFs, NFY subunits and AP2-like factors. This kind of regulation is significant for plant development [9], [52]. Thus, it is plausible to assume that the conservation of these miRNAs and their targets is greatly associated with basic functions for normal growth and development of plants, and could be mobilized to adaptive responses to stress when growth and development are stalled under adverse circumstances. Thus, we can infer the functions of some miRNAs in eggplant based on the functions known in other plants, as these miRNAs have been reported to remain functionally conserved during plant diversification [51].

### Putative Novel miRNAs in Eggplant

High-throughput sequencing technology and whole-genome-scale data mining have enabled and enhanced the discovery of miRNAs in plants. Some miRNAs have not been reported in other plants before, possibly because they are not expressed in normal growth and development, or their expression levels are low and need deeper sequencing to be discovered. Despite lack of genomic sequences from eggplant, the available ESTs helped us to identify two novel miRNAs, m0001 and m0002. Although these predicted miRNAs satisfied most of the criteria, they still require further investigation to fully verify their nature. As non-conserved miRNAs are often expressed at a lower level than conserved miRNAs, our results imply that m0002 is a non-conserved miRNA [53].

BLASTN against eggplant ESTs showed that m0001 matched an *I2-*derived pseudogene, while m0002 matched a protein-coding gene of ARF. The generation of pseudogene-derived sRNAs depends on RDR2, while protein-coding gene-derived sRNAs are generated by several distinct pathways. The *I2* pseudogene is derived from the resistance gene (R gene) *I2* which has been identified in many *Solanum* species [54] and encodes an NBS-LRR protein that confers resistance to vascular wilt disease caused by *Fusarium oxysporum* or *Phytophthora infestans* [55]. The evolution of R genes allows plants to generate novel resistance to match changing patterns of pathogen virulence [56]. Notably, bra-miR1885 was reported to be a newly discovered miRNAs that derived from an R gene, which could be induced specifically by *Turnip mosaic virus* (TuMV) infection [57], [58]. Bra-miR1885 originated through inverted duplication events from protein-coding disease-resistance genes of the TIR-NBS-LRR class, which became bra-miR1885 targets [58]. Similarly, as m0001 derived from a pseudogene of R gene, we may conclude that this new miRNA might play a role in Verticillium wilt response of eggplant. However, the expression of m0001 did not show significant change at 12 h treatment, which indicates that it may not play a role at this stage of pathogen infection.

### The Verticillium Wilt-responsive sRNAs and their Targets in Eggplant

It has been reported that plant disease resistance gene families are comprised of hundreds of members, which are usually targeted by sRNAs [58]–[60]. Since little sRNA information is available for eggplant, a global survey of sRNAs in eggplant seedlings with and without *Verticillium dahliae* infection will enhance our understanding of the regulatory mechanisms of eggplant Verticillium wilt, and provide useful information for improving the Verticillium wilt resistance of economically important crops.

Comparison of the two sequence libraries’ data showed that the size distributions of sRNAs were strikingly different. The CK distribution had a high concentration in 21-nt sRNAs, but the TR distribution was skewed towards 24-nt sRNAs. This may indicate that 24-nt sRNAs were induced by *Verticillium dahliae*, whereas the 21-nt sRNAs were suppressed. The same phenomenon has also been observed in *Verticillium*-inoculated cotton roots [35]. The 21-nt miRNAs and 24-nt miRNAs were sorted into AGO1 and AGO4 clade proteins, respectively. AGO4 was shown to play a role in non-host resistance, basal defense, and effecter-triggered immunity against bacterial pathogens [18]. In our study, more 24-nt miRNAs were obtained in the TR library, which indicated that an AGO4-involved pathway was induced after *Verticillium dahliae* infection, while the AGO1-involved pathway was suppressed. In contrast to 21-nt miRNAs which directly target mRNAs for cleavage, experimental data showed that a 24-nt miRNA could act to direct DNA methylation of their target genes within an 80-nt region around the target sites in association with AGO4 clade proteins [61]. The distribution of kinds of sRNA classes showed the regulatory basis of epigenetic adjustment. Previous studies on several *Arabidopsis* mutants which were implicated in different RNA-silencing pathways suggested that the alteration of *Verticillium* susceptibility is not due to one single RNA-silencing pathway [20]. The different distributions of 21-nt and 24-nt sRNAs between the two libraries provided explicit evidence to support the notion that cross-interaction of multiple RNA-silencing pathways is involved in *Verticillium*-defense response.

After analyzing the 7,716,328 sRNAs obtained from high-throughput sequencing in our study, 99 miRNA families were identified by comparison with known miRNA data in miRBase 19. One third of these miRNA families exhibited altered expression after infection with *Verticillium dahliae*. Among them, 6 miRNA families showed significant changes. These pathogen-regulated miRNAs might contribute to species-specific regulation and act as ‘early’ regulators of signal transduction in stress response. MiR399 and miR395, which are highly induced in nutrition stress [62], were the most strongly down-regulated miRNA families, indicating that they also played an important role in plant defense, but this requires further experimental study. Some highly-conserved, pathogen-responsive miRNAs including miR393, miR160 and miR167, play important roles in regulating perception and signaling of auxin, an important plant hormone that has a central role in plant growth, development, and environmental responses. MiR393 down-regulates *TIR1*, *AFB2*, and *AFB3* transcripts and represses *AFB1* transcription [15] while miR160 and miR167 down-regulate five different ARF transcripts by guiding the cleavage of their cognate mRNAs. It was demonstrated previously that many types of stresses, including bacterial infection, could up-regulate miR393 and repress auxin signaling by keeping TIR1 levels low, thereby increasing AUX/IAA-ARF heterodimerization [63]. However, one interesting observation in our study is that the expression pattern of miR393 was significantly decreased upon *Verticillium* infection, which we also confirmed by stem-loop RT-PCR. The other two miRNAs (miR160 and miR167) involved in regulating auxin signaling pathway also decreased slightly. Similar results could also be found in two recent studies on miRNA responses to fungi infection. Xin et al. [64] investigated the differences in miRNA expression between two wheat cultivars, the disease- susceptible Jingdong8 (JD8) and its near-isogenic resistant line Jingdong8-*Pm30* (JD8-*Pm30*), in response to powdery mildew. They found different miR393 expression patterns between the two near-isogenic lines, with JD8 having an increased level of miR393 while JD8-Pm30 showed a decreased level. Another example also revealed that the *Verticillium*-tolerant cotton cultivar, “Hai-cultivar” had much lower miR393 expression level than the *Verticillium*-sensitive cultivar “Yi-11”, when inoculated with *Verticillium* [35]. In addition, a recent study on *Arabidopsis* showed that inhibition of auxin signal increased susceptibility to the necrotrophic fungi *Plectosphaerella cucumerina* and *Botrytis cinerea* [65]. Further, we examined the expression profile of *TIR1*, an identified target gene of miR393, which is a receptor of auxin, and showed a negative correlation with changes in miR393. Llorente et al. [65] reported that increased expression of *TIR1* leads to enhanced removal of members of the AUX/IAA family of transcription factor (TF) repressors by the SCF E3-ubiquitin ligase proteasome pathway and caused expression of auxin-responsive genes, which, in turn, positively regulate plant resistance to necrotrophic fungi. Interestingly, we found that expression of the ubiquitin-conjugating enzyme E2 gene, an identified target of miR399 was also increased, which may also contribute to the degradation of AUX/IAA. Thus, the decreased expression level of miR393 in *Verticillium*-infected eggplant leads us to speculate that auxin pathways affected by miRNAs could play important roles in resistance to fungal disease. To test this hypothesis, we pretreated eggplant seedlings with different concentrations of NPA and SA which can inhibit the auxin signaling pathway. Our results demonstrated that seedlings pretreated with different concentrations of NPA and SA were more susceptible to *Verticillium dahliae* infection compared to un-pretreated seedlings. These results may partly indicate why miR393 shows down-regulation after *Verticillium dahliae* infection. However, seedlings pretreated with different concentrations of auxin showed less change in plant susceptibility to *Verticillium dahliae*. Further studies will be needed to examine the role of miR393 in fungal infection resistance.

In summary, global transcriptional profiles of small non-coding RNAs were investigated in eggplant seedlings with and without *Verticillium dahliae* infection. The isolated miRNA from eggplant will help us to identify the miRNA-based regulatory system in this species. The differential patterns of sRNA expression are a valuable resource for further studies on post-transcriptional gene regulation in the defense response of eggplant to Verticillium wilt. Hence, further identification and detailed analysis of the target genes of these sRNAs will deepen our understanding of their regulatory roles in this pathological response, and uncover the mechanisms of *Verticillium* infection.

## Supporting Information

Figure S1 **Stem loop structure of the novel miRNA m0001 (A) and m0002 (B) precursors.**
(TIF)

Figure S2 **Scatter plot of miRNA differential expression between CK and TR libraries.** Red plots represent miRNAs with increased expression; green plots represent miRNAs with reduced expression; blue plots represent equally-expressed miRNAs.
(TIF)

Table S1 **RT-PCR primer sequences.**
(DOC)

Table S2 **Evolutionary conservation of known eggplant miRNAs.**
(XLS)

Table S3 **Predicted targets of the identified known and novel miRNAs.**
(XLS)

Table S4 **Summary of common and specific sequences between CK and TR.**
(DOC)

Table S5 **Differentially expressed miRNAs between libraries.**
(XLS)
