# Supplementary figures and images for: Identification of MiRNA from Eggplant (Solanum melongena L.) by Small RNA Deep Sequencing and Their Response to Verticillium dahliae Infection
Source: PLoS One. 2013 Aug 27;8(8):e72840. doi: 10.1371/journal.pone.0072840 (PMC3754920; doi:10.1371/journal.pone.0072840)

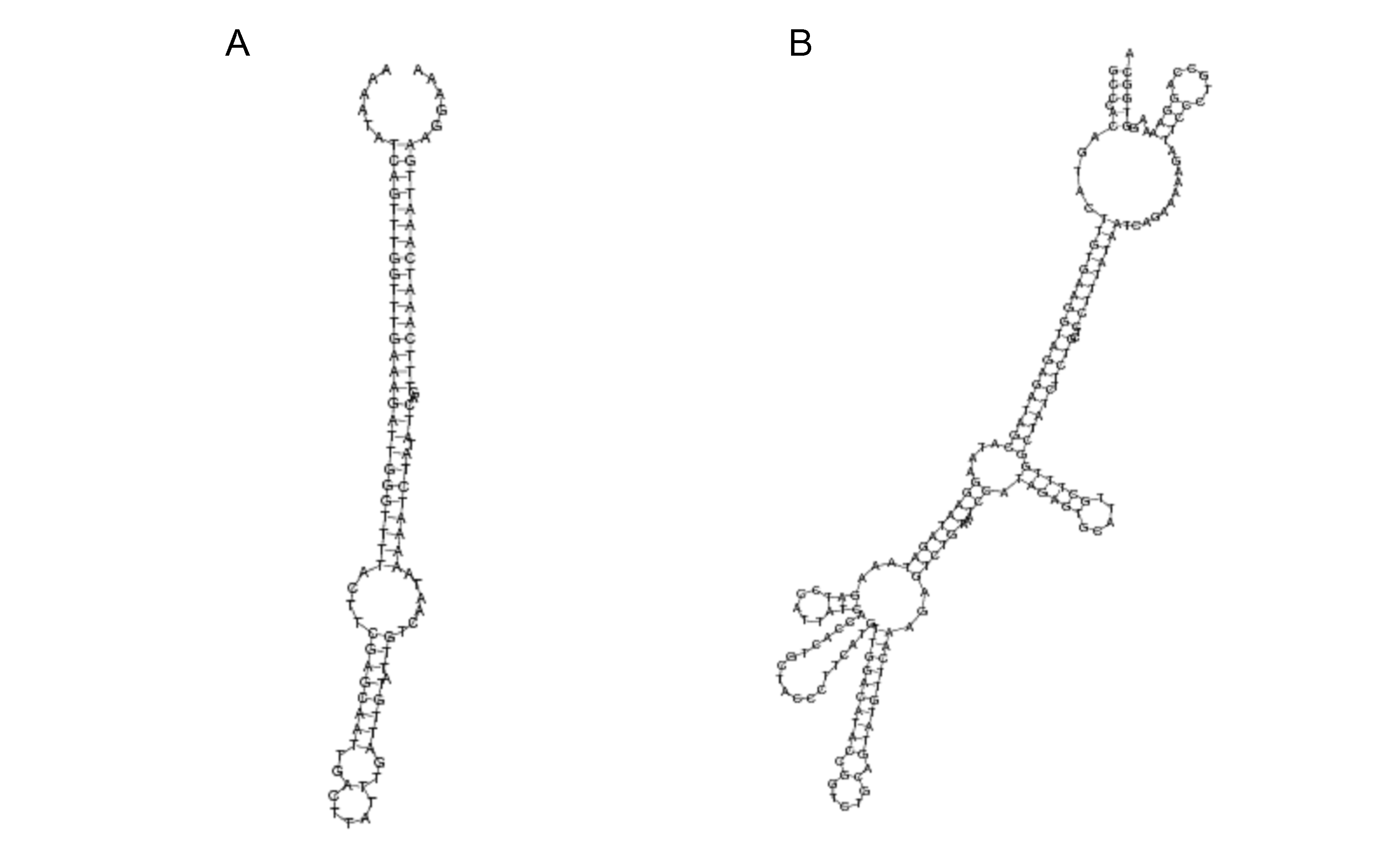

Supplement: Figure S1 — Stem loop structure of the novel miRNA m0001 (A) and m0002 (B) precursors. (TIF) [file pone.0072840.s001.tif]

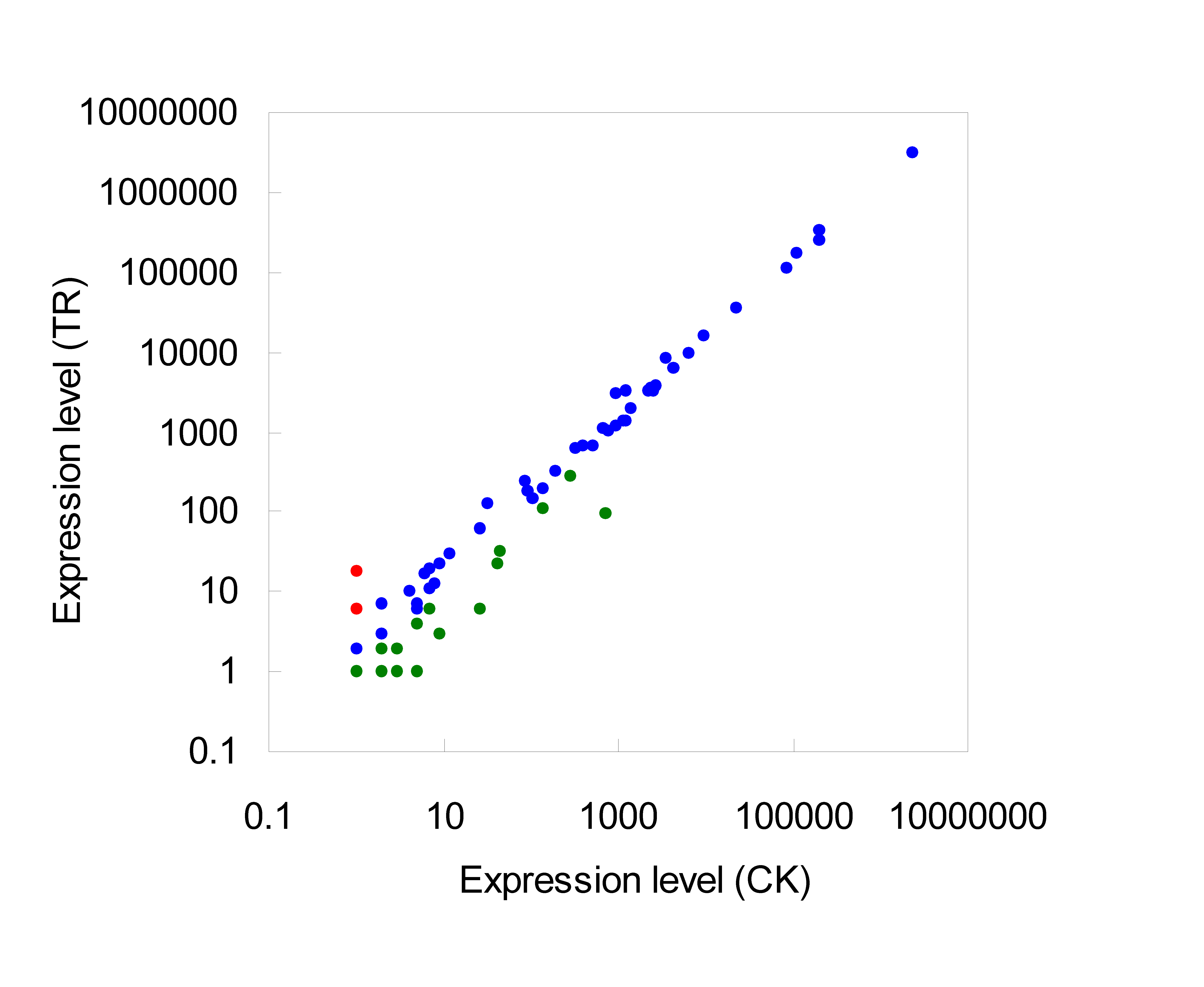

Supplement: Figure S2 — Scatter plot of miRNA differential expression between CK and TR libraries. Red plots represent miRNAs with increased expression; green plots represent miRNAs with reduced expression; blue plots represent equally-expressed miRNAs. (TIF) [file pone.0072840.s002.tif]
