# Supplementary material for: Identification of MiRNA from Eggplant (Solanum melongena L.) by Small RNA Deep Sequencing and Their Response to Verticillium dahliae Infection
Source: PLoS One. 2013 Aug 27;8(8):e72840. doi: 10.1371/journal.pone.0072840 (PMC3754920; doi:10.1371/journal.pone.0072840)
Supplement: Table S1 — RT-PCR primer sequences. (DOC) [file pone.0072840.s003.doc]

**Table S1.** **RT-PCR** **primer sequences**

| Primer name | Primer sequences |
| --- | --- |
| 156_RT | GTCGTATCCAGTGCAGGGTCCGAGGTATTCGCACTGGATACGAGTGCTC |
| 156_F | ATGCCCGCTGACAGAAGA |
| 393_RT | GTCGTATCCAGTGCAGGGTCCGAGGTATTCGCACTGGATACGAATTCCG |
| 393_F | AGCCCATCATGCGATCT |
| 395_RT | GTCGTATCCAGTGCAGGGTCCGAGGTATTCGCACTGGATACGAGAGTTC |
| 395_F | GGTGAGGCTGAAGTGTTTG |
| 399_RT | GTCGTATCCAGTGCAGGGTCCGAGGTATTCGCACTGGATACGATAGGGC |
| 399_F | CAATGCCGCCAAAGG |
| 482_RT | CTCAACTGGTGTCGTGGAGTCCCCCAATTCAGTTGAGGGCATGG |
| 482_F | GCCCGCTCTTGCCTGC |
| m0001_RT | GTCGTATCCAGTGCAGGGTCCGAGGTATTCGCACTGGATACGAAACCCA |
| m0001_F | TGTCCCGTTTGGTTTGA |
| m0002_RT | GTCGTATCCAGTGCAGGGTCCGAGGTATTCGCACTGGATACGATGCTAT |
| m0002_F | GCTTGGCTTGTGAAGGTAG |
| Uni_R | GTGCAGGGTCCGAGGT |
| U6_F | CGGGGACATCCGATAAAA |
| U6_R | TTGGACCATTTCTCGATTTG |
| TIR1_F | AGTCGCTCAACAAAGGGTAA |
| TIR1_R | ACAGGACAGTAGCAGGAAGG |
| TC4976_F | TGAGGAGCCACAAAGGGA |
| TC4976_R | TCATCAACAAGGGCAACACT |
| FS072237_F | GCCTGCTCCAAAGTCATC |
| FS072237_R | CACCTATAACGGAAGAAGAAAT |
| PHO2_F | GCACTTCTACAAGGACCACA |
| PHO2_R | AGTTCCAACAACGACCGA |
| FS082687_F | GCTGATAGGATTAGGGATGA |
| FS082687_R | GACTCGAAAGATTGGGAAG |
| TC14175_F | CTCATTTTACCTCTAATCCTCC |
| TC14175_R | AAAACAGTCATGCCTTGGT |
| TC5469_F | TTTCCTCTTTACTGCCACCTT |
| TC5469_R | GGGATACTCATTGCCCACA |
| TC9181_F | TATCTTTTCCCCTGCTTTC |
| TC9181_R | CGTAGGCTTACTCCTCACC |
| EF-1α_F | ATTCAAGTATGCCTGGGTGCT |
| EF-1α_R | GTGGTGGAGTCAATAATGAGGAC |
