# Supplementary material for: Identification of MiRNA from Eggplant (Solanum melongena L.) by Small RNA Deep Sequencing and Their Response to Verticillium dahliae Infection
Source: PLoS One. 2013 Aug 27;8(8):e72840. doi: 10.1371/journal.pone.0072840 (PMC3754920; doi:10.1371/journal.pone.0072840)
Supplement: Table S4 — Summary of common and specific sequences between CK and TR. (DOC) [file pone.0072840.s006.doc]

Table S4 Summary of common and specific sequences between CK and TR

|  | Unique sRNAs | Percentage (%) | Total sRNAs | Percentage (%) |
| --- | --- | --- | --- | --- |
| Total sRNAs | 7,716,328 | 100% | 30,830,792 | 100% |
| TR & CK | 1,016,641 | 13.18% | 22,666,440 | 73.52% |
| TR specific | 4,956,483 | 64.23% | 6,297,580 | 20.43% |
| CK specific | 1,743,204 | 22.59% | 1,866,772 | 6.05% |
